# Macamides as Potential Therapeutic Agents in Neurological Disorders

**DOI:** 10.3390/neurolint16060117

**Published:** 2024-11-21

**Authors:** Karin J. Vera-López, Gonzalo Davila-Del-Carpio, Rita Nieto-Montesinos

**Affiliations:** Escuela Profesional de Farmacía y Bioquímica, Universidad Católica de Santa María, Urb. San José s/n—Umacollo, Arequipa 04000, Peru; kvera@ucsm.edu.pe (K.J.V.-L.); gdavilad@ucsm.edu.pe (G.D.-D.-C.)

**Keywords:** macamides, therapeutic agents, neurological diseases, agonist function

## Abstract

Therapeutic treatment of nervous system disorders has represented one of the significant challenges in medicine for the past several decades. Technological and medical advances have made it possible to recognize different neurological disorders, which has led to more precise identification of potential therapeutic targets, in turn leading to research into developing drugs aimed at these disorders. In this sense, recent years have seen an increase in exploration of the therapeutic effects of various metabolites extracted from Maca (Lepidium meyenii), a plant native to the central alpine region of Peru. Among the most important secondary metabolites contained in this plant are macamides, molecules derived from N-benzylamides of long-chain fatty acids. Macamides have been proposed as active drugs to treat some neurological disorders. Their excellent human tolerance and low toxicity along with neuroprotective, immune-enhancing, and and antioxidant properties make them ideal for exploration as therapeutic agents. In this review, we have compiled information from various studies on macamides, along with theories about the metabolic pathways on which they act.

## 1. Introduction

According to a study by the United Nations Population Division published in 2022 [1], the global population’s life expectancy has increased to 72.3 years. The same report mentions that if current levels of increase continue, the average lifespan will reach 77.2 years by 2050. This increase in longevity implies greater challenges to healthcare systems due to age-related declines in physiological functioning. Among those conditions that most affect adults are neurological diseases and disorders, which today are a leading cause of disability and the second-leading cause of mortality worldwide [2,3]. In Perú, the mortality rate due to neurological conditions is only 44% of that registered globally (61.1 and 139, respectively) per 100,000 people; however, regarding the prevalence rate, the number of registered cases is very close (35,334.6 versus 41,204.1), representing a difference of only 15% from the same population sample [4].

**Figure 1:**
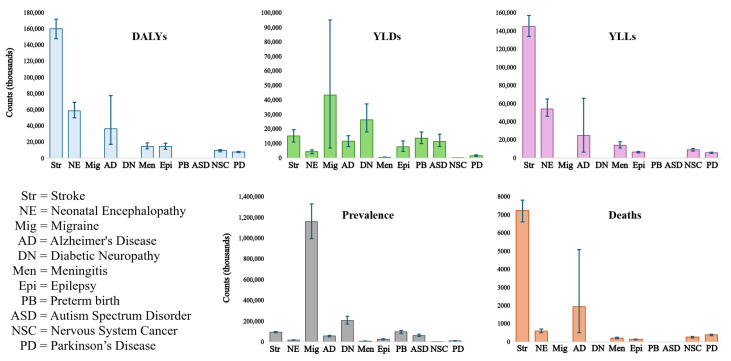
Error bar plots of the measures used to obtain the leading neurological disorders’ global, regional, and national burden [4]; the 95% uncertainty intervals are depicted as blue bars.

Classifying neurological diseases is challenging due to their multifactorial nature [5]. Today, it is known that there are more than 600 diseases related to neurological deterioration [6]. Nevertheless, according to Steinmetz et al., 37 conditions are considered the leading cause of disability-adjusted life years (DALYs), affecting almost 3.5 billion people worldwide [4]. Among these conditions, the top ten account for 67.8% of the total DALYs reported by the authors, with stroke being the primary reason (see Table 1). Although Parkinson’s disease (PD) does not appear in the top ten, it is the tenth-most prevalent neurological disorder in the Andean region of Latin America, where Peru is located, as well as the fourth-leading cause of death, above epilepsy, meningitis, and cancer of the nervous system (Table 1 and Figure 1). It is worth noting that among these conditions, four are the most common due to age: Alzheimer’s disease (AD), Parkinson’s disease, stroke, and epilepsy [6].

Despite advancements in medical science and technology, developing effective treatments for neurological disorders remains one of the most complex and demanding tasks in drug approval. Unfortunately, the failure rate of new therapies targeting these disorders exceeds that of treatments for non-neurological conditions [7]. Because of this, it is crucial to explore new therapeutic approaches and drug development tools directed at more than the temporary relief of disease symptomatology. Many novel strategies today are being driven toward the more than 330 non-sensory G-protein-coupled receptors (GPCRs) expressed in the brain. GPCRs involve numerous physiological functions, mainly endocrine and neurological processes [3,8].

GPCRs constitute the most important family of cell surface receptors, being expressed by about 4% of the human genome [9]. With more than 800 members, these receptors are crucial for transmitting extracellular signals and regulating mammalian physiological processes in various organic systems [10]. In the nervous system, for example, they are responsible for synaptic plasticity by regulating neurotransmission in pre- and postsynaptic sites, neurogenesis, immune and behavioral regulation, cognitive functions, and pain, among many others [3,9,11,12]. Structurally, GPCRs are transmembrane proteins that consist of seven helical regions linked by six loop-shaped structures. Three of these structures are found in the extracellular part containing the N-terminal domain, while the remaining three are located in the cytoplasmic region with the C-terminal domain. These receptors have unique ligand-binding pockets that allow them to interact with various stimuli [13]. The specificity of GPCRs is so high that it allows the nervous system to filter and select specific signals that will trigger a series of responses within the cell [14]. GPCRs facilitate signal transduction through arrestins and heterotrimeric G proteins. Understanding GPCR functions in the nervous system and disease mechanisms could accelerate drug development [15].

Leading pharmaceutical companies spend billions of dollars researching and developing new drugs with these receptors as therapeutic targets. Among the promising novel medicines in treating neurological diseases are macamides [16,17,18,19]. Extracted from Maca (*Lepidium meyenii*), macamides are non-polar secondary metabolites with a structure composed of an N-benzyl ring linked to a long chain of fatty acids through an amide bond. The Maca plant thrives in extreme high-altitude conditions (over 4000 m) [20], including cold temperatures, intense ultraviolet radiation, and low air pressure [16]. Currently, Maca is sold from Peru to North America and Europe for its nutritional and functional benefits [21]. These benefits include fertility-enhancing, neuro-protection, antioxidant, anti-inflammatory, anti-osteoporosis, antiviral, aphrodisiac, immunostimulant, and anabolic or hormonal balancing properties. Moreover, regular intake of Maca has been shown to slow the progression of chronic neurodegenerative disorders such as Alzheimer’s disease (AD) [22,23], Parkinson’s disease (PD) [17], and Huntington’s disease (HD) [24].

Exploring new therapeutic agents in the treatment of neurological diseases is of vital importance to improving the quality of life of people who suffer from them. Macamide molecules have been recognized for their neuroprotective effects, and have become drugs of potential interest to the pharmaceutical industry. This work aims to collect the most recent information about research involving this family of Maca derivatives in treating three diseases related to neurological conditions (Alzheimer’s disease, stroke, and Parkinson’s disease) and involving GPCRs as therapeutic targets. For this review, we have considered the last five years (2019 to the date of publication) in our search for papers related to this topic.

## 2. Macamides as Potential Drugs Affecting GPCRs

According to the World Health Organization, neurological diseases are responsible for nine million deaths per year worldwide [2]. In addition, one in three people will develop a disorder related to neurological disease at some point in their lives. The economic burden represented by neurological disorders is enormous, and the indirect costs and costs of disability are often greater than those involved in medical care. For example, it is estimated that in Latin America, governments allocate less than 2% of their health budgets to treating these disorders [25]. In some Central American countries these resources do not even exceed three US dollars per person [26]. Excessive cost of treatments, lack of information, stigmatization, and deficiency in health programs are all parts of the problem that urgently need to be addressed.

Different strategies have been designed to address this problem, with the development of new drugs being one of the fields with the most remarkable growth [3,4,6]. One family of bioactive components being studied for their therapeutic and neuroprotective properties is the macamides [27,28]. Extracted mainly from the tuberous roots of Maca, these secondary metabolites have been part of the diet of numerous populations in the Peruvian highlands of the Central Andes mountain range since ancient times [29]. Numerous studies have been carried out on the therapeutic effect of macamides in treating and preventing different diseases such as osteoporosis [30,31], cancer [32,33,34], cardiovascular disease [35], and cellular damage [19]. According to work by Zhu et al. [16], there are about 26 macamides considered as representative components of Maca, many of which are considered potential therapeutic agents (Table 2).

**Figure 2:**
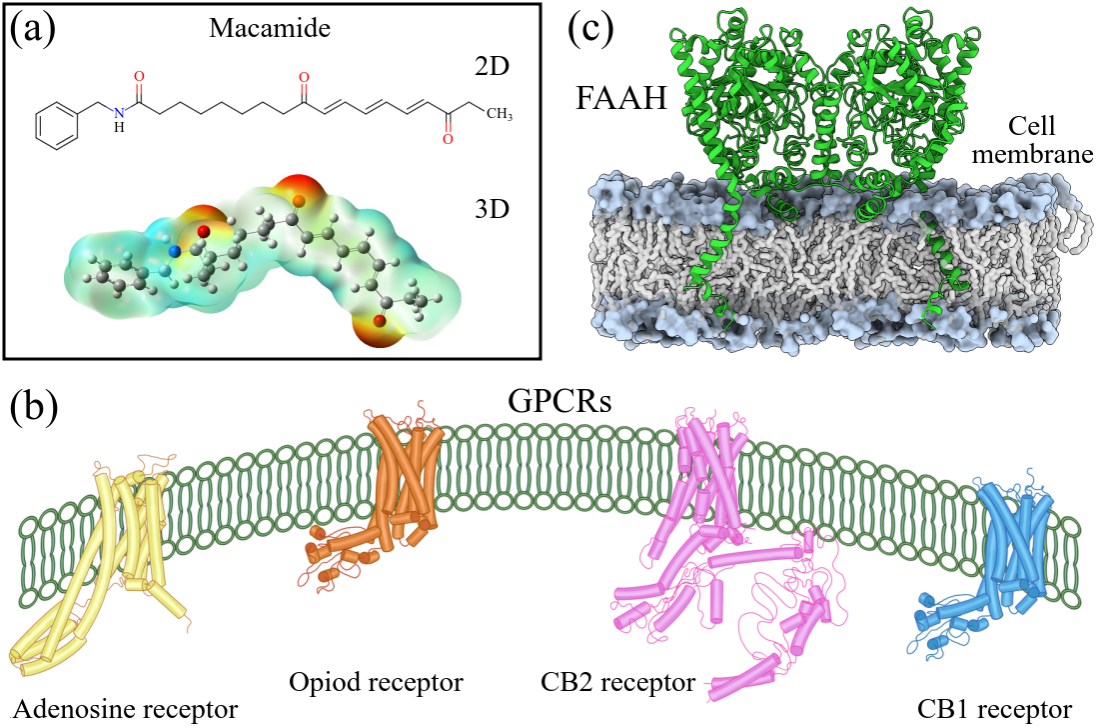
Macamides as potential drugs in the treatment of neurological diseases: (**a**) 2D and 3D structure of macamide MM01 ([16]), where the 3D structure shows the electrostatic surface calculated at a B3LYP/3ZVP level of theory, with red, blue, and green indicating nucleophilic zones, electrophilic zones, and neutral zones, respectively; (**b**) structure of the fatty acid amide hydrolase 1 (FAAH) enzyme involved in the degradation of anandamide, a neurotransmitter related to the CB1 receptors of the nervous system and CB2 of the peripheral nervous system; (**c**) schematic representation of the Adenosine A2A, opioid, CB2, and CB1 receptors, which are involved in different neurological disorders.

Structurally, macamides share a common group or core, which can be a benzylamine group (Ph-CH_2_-NH-CO-) or its derivative m-methoxybenzylamine (CH_3_-O-Ph-CH_2_-NH-CO-) [30,36]. The main difference between these metabolites lies in the group attached to this core, which can be an aliphatic chain or a fatty acid with different degrees of unsaturation. In general, macamides are mostly made up of alkyl groups, which gives their structure a hydrophobic character (Figure 2a). However, they have one or more carbonyl groups (C=O), which introduces polar regions to the molecule that can be electron donors. Several authors have mentioned that this amphipathic character of macamides could be responsible for their antioxidant properties and ability to bind to other molecules, including cellular receptors, among them GPCRs [18,30,37,38,39,40].

Currently, four GPCRs have been recognized in which macamides can directly affect the biological functions (Figure 2b). Among thees, the best known are the CB1 and CB2 receptors, which belong to the endocannabinoid system and are responsible for intercellular communication, release of neurotransmitters, and regulation of pain perception, among several other functions [17,18,35]. A third receptor is adenosine A2A, one of the receptors responsible for regulating the cardiovascular and immune systems and neurotransmitter secretion of neurotransmitters [38,41]. Finally, the fourth receptor consists of the mu opioid receptor (MOR) subtypes, which regulate pain, mood, memory, and locomotion, among other functions [42,43].

### 2.1. CB1 y CB2 Receptors

Discovered in 1988 and 1993, respectively, the CB1 (cannabinoid receptor type 1 [44]) and CB2 (cannabinoid receptor type 2 [45]) receptors belong to the endocannabinoid system, which is composed of receptors and endocannabinoids. These latter are endogenous ligands of lipid nature [46]. CB1 receptors are the most abundant metabotropic receptors in the brain. They are highly expressed in the hippocampus, basal ganglia, cortex, and cerebellum and minorly expressed in other brain areas [47,48]. They are also found in peripheral organs such as the adipocytes, liver, lungs, and immune system [49]. In the case of CB2 receptors, their distribution is mainly restricted to the periphery in cells of the immune system, nerve fibers of the skin, bone cells, liver cells, and pancreas [45,49,50]. These receptors have also been found in the central nervous system. Evidence suggests that CB2 receptors play a role in emotional behaviors and that they may be present in neurons or glial cells [51,52].

When these receptors are activated, they can control the release of neurotransmitters in the brain and other parts of the nervous system [46]. This activation can affect functions such as pain perception, mood, appetite, and memory. At the molecular level, activating these receptors triggers a series of signals inside cells that regulate cellular activity and communication between neurons [35,46]. The extensive distribution in the body of CB1 and CB2 receptors explains why cannabinoids can affect different bodily functions differently.

One of the most studied processes of the endocannabinoid system is the role that the fatty acid amide hydrolase (FAAH) enzyme has in the degradation of anandamide or arachidonoylethanolamide (AEA) (Figure 2c), one of the main endocannabinoids synthesized by the body [18,53]. AEA is a neuromodulator through its agonist or activator effect on CB1 and CB2 receptors. In addition, this endocannabinoid is vital in creating memory and sensations such as hunger, sleep patterns, and pain relief. In their in vivo experiments, Vázquez et al. showed that exogenous anandamide administration reduced neuronal damage and acted as a brain protector from acute injury by promoting an enhanced proinflammatory glial response in the brain [54]. These results indicate that endocannabinoids could positively treat neurological diseases and other pathologies. Several of these inhibitors are currently being investigated in this regard [18].

In this context, Alasmari et al. worked on the ability of four macamides to inhibit the action of FAAH: N-benzylstearamide, N-benzyloleamide, N-benzyl-9Z,12Z-octadecadienamide, and N-benzyl-9Z,12Z,15Z-octadecatrienamide [18]. Using in vitro techniques, their results exhibited that these four macamides had different levels of inhibitory effectiveness, with N-benzyl-9Z,12Z-octadecadienamide showing the highest FAAH inhibitory activity and N-benzylstearamide the lowest. Remarkably, this inhibitory activity was shown to be nonreversible. In another study, Hajdu et al. demonstrated that the macamide N-benzyl-9Z,12Z-octadecadenamide has sub-micromolar and selective binding affinities for the CB1 receptor. Furthermore, it also exhibited weak inhibition of FAAH and potent inhibition of anandamide cellular uptake [55]. In 2020, Singh et al. showed that macamides could inhibit the degradative action of the soluble enzyme epoxy hydrolase (sEH) due to its similarity with the structure of epoxy fatty acids (EpFA) [56]. Performing in vitro tests with nineteen macamides synthesized for their work and in vivo tests with two, they observed that the macamide N-benzyl-9Z,12Z-octadecadienamide (N-Benzyl-linoleamide) had an inhibitory action on sEH. Based on their results, they suggest that macamides could act as dual inhibitors of sEH and FAAH, producing a synergistic effect. This would translate into advantages over other therapies due to its direct interaction with the CB1 receptor, which could provide new therapies aimed at neurological diseases. Furthermore, activation of the CB1 receptor by macamides could have several physiological effects, as this receptor regulates pain perception, appetite, mood, and memory [27,30,35]. Therefore, macamides and other compounds that interact with the CB1 receptor have the potential to significantly affect the function of the nervous system and other body systems. From a therapeutic point of view, this interaction could be relevant for developing treatments for various medical conditions in which modulation of the endocannabinoid system and the CB1 receptor may be beneficial. However, it is essential to continue this research in order to better understand the specific effects of macamides on CB1 receptor activation and their implications for human health.

### 2.2. Adenosine Receptor

Adenosine is a natural compound found in mammalian tissues that regulates various functions in the brain [57]. In the central nervous system (CNS), adenosine controls neuronal excitability and modulates the activity of astrocytes and microglia [58]. Adenosine binds to four receptor subtypes: A_1_, A_2*A*_, A_2*B*_, and A_3_. Among these receptor subtypes, A_2*A*_ is a promising drug target because it is involved in various pathological conditions, including neurological diseases [59,60]. Moreover, the A2A receptor plays essential roles in regulating glutamate and dopamine release, making it a potential therapeutic target for conditions such as insomnia, pain, depression, and fatigue [38]. Additionally, caffeine, a widely consumed substance, exerts its stimulant effects primarily by blocking A_2*A*_s [41,58]. In the CNS, A_2*A*_ is expressed in striatopallidal medium spiny neurons [58,61]. A_2*A*_ receptors are located in neurons (presynaptically and postsynaptically), astrocytes, microglia, oligodendrocytes, and capillary endothelial cells at the cellular level. A_2*A*_ is a promising drug target for various pathological conditions. Research has shown that blocking adenosine A_2*A*_ receptors can improve motor dysfunction in Parkinson’s disease (PD) [61]. Furthermore, adenosine receptors regulate the secretion of neurotransmitters and the cardiovascular, immune, and other major systems [59].

Fatigue is not only a typical limitation of endurance to physical exertion but also a symptom in many neurodegenerative diseases. This symptom, known as central fatigue, impairs everyday activities in patients with multiple sclerosis, Parkinson’s disease, and Alzheimer’s disease [62]. The pharmacological and environmental approach to central fatigue is ineffective because the pathophysiology is unclear. However, increased density of neuronal adenosine A_2*A*_ receptors is associated with these neurodegenerative diseases’ pathology and clinical conditions [63,64].

Recent studies have shown that a diet rich in macamides can improve physical strength, reduce oxidative stress, and prevent skeletal muscle damage and fatigue [65]. Zhu et al. demonstrated that incorporating the macamide N-benzyl-linoleamide into the diet can effectively combat fatigue with few side effects [66,67]. In another study, Liu et al. showed through an analysis of intestinal microbiota in mice that this macamide was metabolized more efficiently than a sample of 26 macamides. Their results showed that its consumption reduced exercise-induced skeletal muscle fatigue in mice by modulating the L-glutamate-ornithine-proline axis through the degradation of histidine, arginine, and proline [38]. The authors determined that the adenosine A_2*A*_ receptor interacted with 16 of the 26 macamides that they analyzed, including N-benzyl-linoleamide.

### 2.3. Opiod Receptor

The opioid receptor is a cellular receptor activated by opioids of either endogenous or synthetic origin. Opioid receptors are located in central and peripheral nervous system neurons and immune cells [68]. Their activation modulates pain, mood, and behavior transmission, exerting analgesic, calming, and rewarding effects. There are four main subtypes of opioid receptors: mu (μ, MOR), delta (δ, DOR), kappa (κ, KOR), and nop (NOR), all of which are members of the GPCR superfamily [42,69]. Each of these receptors has a different physiological function, and they are expressed in different regions of the brain and tissues [68]. Activation of opioid receptors plays a crucial role in signal transduction and in treating pain, cancer, neurodegenerative disorders, and cardiac insults [70].

Hou et al. investigated several important chemical components in these species in order to clarify their analgesic targets and mechanism of action [42]. Using four opioid receptor target models, they evaluated the pharmacological properties of different agonist and antagonist molecules against each subtype model using label-free phenotypic dynamic mass redistribution (DMR) assays. The label-free DMR assay is an advanced technology capable of real-time detection of integrated cellular responses. Their results showed that one compound isolated from Maca had agonistic activity on the mu opioid receptor. Research has shown that certain Maca extracts, including macamides, may modulate opioid receptors. This modulation could have both anxiolytic and antidepressive effects, which would help to improve mood and stress. Mu opioid receptor modulation has also been observed to have analgesic activity; as with adenosine receptors, macamides may also provide synergistic activity with CB1 and CB2 receptors. Such activity could help to treat physiopathies such as pain as well as to improve mood and control appetite.

## 3. Macamides as Potential Drugs for Neurological Diseases

### 3.1. Alzheimer’s Disease

**Figure 3:**
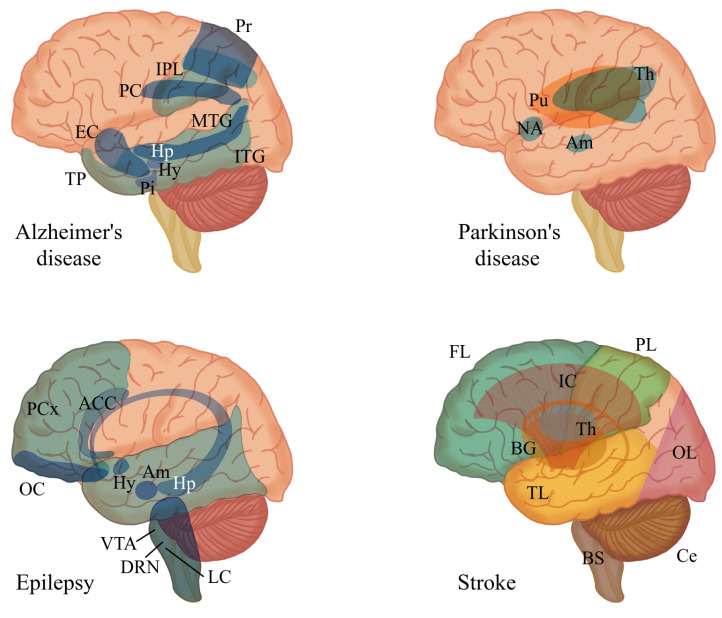
Main brain regions affected by four major neurological disorders: Alzheimer’s disease, Parkinson’s disease [3], epilepsy [79], and stroke [80]. Abbreviations for the specified brain regions are as follows: anterior cingulate cortex (ACC), amygdala (Am), basal ganglia (BG), brain stem (BS), cerebellum (Ce), dorsal raphe nuclei (DRN), entorhinal cortex (EC), frontal lobe (FL), hypothalamus (Hy), hippocampus (Hp), inferior parietal lobule (IPL), inferior temporal gyrus (ITG), internal capsule (IC), locus coeruleus (LC), medial temporal gyrus (MTG), nucleus accumbens (NA), occipital lobe (OL), orbitofrontal cortex (OC), parietal lobe (PL), pituitary (Pi), posterior cingulate (PC), precuneus (Pr), prefrontal cortex (PCx), putamen (Pu), temporal lobe (TB), temporal pole (TP), thalamus (Th), ventral tegmental area (VTA).

Alzheimer’s disease (AD) is a chronic, progressive, and irreversible neurodegenerative disease characterized by the loss of cognitive function (praxis, visuospatial, and executive dysfunction), short- and long-term memory deficits, and negative personality and sociability changes in patients [71,72]. AD is characterized by the accumulation of amyloid-β (Aβ) in amyloid plaques and hyperphosphorylated aggregates of the microtubule-associated tau protein in neurofibrillary tangles, which are first detected in the frontal and temporal lobes and then slowly progress to the other areas of the neocortex (Figure 3) [73,74,75]. Other molecular mechanisms have also been proposed in its pathogenesis, including mitochondrial dysfunction, oxidative stress, neuroinflammation, excitotoxicity, and calcium dysregulation, which cause neurodegeneration by different molecular pathways [76,77,78]. This disease is the most common cause of dementia, reaching rates of around 65% of all dementia cases. Although it is a disease that affects older adults in a more significant proportion, reaching more than 50% over the age of 85, it can also affect young people [72].

Even though the population presenting symptoms of AD is increasing and numerous research works have been carried out to find drugs that can combat the disease, the results have not been very encouraging [81,82]. Today, there are only four drugs approved by the FDA for treating AD: donepezil, galantamine, and rivastigmine, all of which are AChEIs, and the NMDA antagonist memantine [82,83]. However, Xia et al. recently published an in silico and in vivo study evaluating the interaction capacity of 12 macamides with the enzyme acetylcholinesterase (AChE) [84]. This enzyme is one of those responsible for controlling the level of acetylcholine in the hippocampus. ACh is closely associated with learning and memory, while its decrease is associated with age; in the case of AD, it is associated with an increase in amyloid plaques. Their results showed that the macamide N-benzyl-9Z,12Z-octadecadienamide formed very energetically stable complexes with AChE. As a result, it was chosen for further in vivo experiments. Animal assays indicated that this macamide was effective in preventing scopolamine-induced cognitive impairment and neurotransmitter disorders. It also increased the favorable rates of Nrf2 and HO-1 in hippocampal CA1, improved synaptic plasticity by maintaining synaptic morphology, and increased synapse density. Additionally, this macamide reduced the contents of IL-1β, IL-6, and TNF-α in the hippocampus, serum, and colon, promoted colonic epithelial integrity, and partially restored the composition of gut microbiota to normal.

### 3.2. Stroke

A stroke is an acute focal neurological deficit attributed to a vascular lesion (cerebral infarction, intracerebral hemorrhage (ICH), or subarachnoid hemorrhage (SAH)) of the central nervous system [85]. Stroke is the second leading cause of death in the world (5.5 million deaths in 2016) and the leading cause of severe long-term disability, with 116 million disability-adjusted life years [86]. Additionally, one-third of stroke survivors require post-stroke care. Age is considered to be a risk factor in the incidence of stroke [87]. Studies indicate that the probability of suffering a stroke doubles after the age of 45 compared to the population under that age. Regarding the prevalence of cases, it is estimated that between 40 and 60 years of age the prevalence is 1%, increasing to 12% in people over 80. There are three main types of stroke: ischaemic, intracerebral, and subarachnoid hemorrhage [88]. An analysis carried out in the USA on all cases of strokes showed that 87% were of ischemic origin, 10% were due to ICH, and 3% were due to SAH [89]. However, the percentages and causes depend on the population and ethnic group. The leading causes of ischemic strokes include atherosclerosis in the arteries, cardioembolic pathologies such as atrial fibrillation, and occlusion of small vessels.

Neonatal hypoxic–ischemic encephalopathy (HIE) is a common cause of brain injury due to oxygen deprivation and reduced blood flow [28]. It has serious long-term consequences, including cerebral palsy and cognitive and behavioral deficits. While therapeutic hypothermia is a standard treatment, it is insufficient to prevent severe neurodevelopmental disorders. Currently, there is no effective medical treatment for nerve damage caused by HIE. Developing safe and effective neuroprotective therapeutics is crucial for long-term recovery. In this regard, Jiao et al. evaluated the neuroprotective capacity of the macamide N-benzyl eicosapentaenamide (NB-EPA) in hypoxic–ischemic brain injuries [28]. Their trials conducted in neonatal mice showed that this macamide improved hypoxic–ischemic brain damage by alleviating infarct size and improving neurobehavioral disorders. Furthermore, NB-EPA inhibited neuronal apoptosis, enhancing neuron survival and proliferation by activating phosphorylated AKT signaling. In this same line of research, Yang et al. investigated possible neuroprotective mechanisms of macamides on neonatal hypoxic–ischemic brain damage (HIBD) [19]. For this purpose, they used macamide B (N-benzyl-9Z,12Z-octadecadienamide) and explored its effect on autophagy and apoptosis induced by HIBD. Their results were remarkable, indicating that this macamide significantly reduced brain damage and improved neuronal recovery.

## 4. Conclusions

The therapeutic properties of macamides are still under continuous evaluation; however, numerous research groups have shown that this family of metabolites have potential uses in the prevention and treatment of various diseases. Macamides are natural origin, and it has been shown that their consumption does not present side effects. In addition, they have essential advantages in health care due to their antioxidant and antiosteoporotic properties, as well as above all their neuroprotective activity, which has the potential to positively affect vulnerable populations such as older adults. In this work, we present the latest research from a qualitative approach into macamides for use as neuroprotective agents. We highlight their role in treating three of the leading neurological diseases with the highest incidence, namely, Alzheimer’s disease, stroke, and Parkinson’s disease.

The findings could not be more promising. Macamides have been shown to have agonist activity and to modulate the function of GPCRs associated with neurological disorders. Of particular note are several studies showing that the macamide N-benzyl-9Z,12Z-octadecadienamide has a high affinity with GPCRs and exerts therapeutic effects in all three diseases we analyzed.

There are still many areas to be investigated and many aspects to be analyzed among the mechanisms that involve macamides and their beneficial effects in the neuropathologies of various diseases, especially from broader and more quantitative approaches. Studies such as meta-analyses could be appropriate in future research. However, we hope that this review, while limited in some respects, can be helpful to groups specializing in developing drugs and treatments.

## Figures and Tables

**Table 1:** Top ten neurological conditions leading to the most nervous system DALYs in 2021.

^a^ Position	Neurological	DALYs	YLDs	YLLs	Prevalence	Deaths
	Conditions					
1 (1)	Stroke	160,000	15,200	145,000	93,800	7250
		[148,000 to 172,000]	[11,000 to 19,400]	[134,000 to 157,000]	[89,000 to 99,300]	[6610 to 7820]
2 (3)	Neonatal encephalopathy	58,600	4280	54,300	18,600	604
		[50,100 to 69,000]	[3100 to 5590]	[46,000 to 64,900]	[16,100 to 21,100]	[511 to 722]
3 (2)	Migraine	-	43,400	-	1,160,000	-
			[6740 to 95,100]		[996,000 to 1,330,000]	
4 (5)	^b^ Alzheimer’s disease	36,300	11,600	24,700	56,900	1950
		[17,200 to 77,400]	[7960 to 15,300]	[6370 to 65,700]	[49,400 to 65,000]	[503 to 5080]
5 (4)	Diabetic neuropathy	-	26,300	-	206,000	-
			[18,000 to 37,400]		[171,000 to 249,000]	
6 (19)	Meningitis	14,500	603	13,900	7270	214
		[11,500 to 18,700]	[425 to 791]	[11,000 to 18,000]	[5930 to 9070]	[177 to 266]
7 (6)	Epilepsy	14,400	7760	6610	24,400	140
		[11,000 to 18,500]	[4660 to 11,800]	[5450 to 7340]	[18,600 to 30,800]	[116 to 153]
8 (9)	^c^ Preterm birth	-	13,800	-	97,500	-
			[9950 to 17,900]		[83,000 to 112,000]	
9 (8)	autism spectrum disorder	-	11,500	-	61,800	-
			[7840 to 16,300]		[52,100 to 72,700]	
10 (7)	nervous system cancer	9200	132	9070	1030	264
		[7890 to 10,600]	[93.8 to 174]	[7750 to 10,500]	[907 to 1140]	[226 to 302]
11 (10)	Parkinson’s disease	7470	1670	5800	11,800	388
		[6730 to 8140]	[1170 to 2210]	[5250 to 6260]	[10,400 to 13,400]	[345 to 419]

All data extracted from [4]. ^a^ Neurological conditions ordered according to the global ranking; the numbers in parentheses correspond to the ranking for the Andean Latin America region. DALYs = disability-adjusted life years; YLDs = years lived with disability; YLLs = years of life lost. Values are expressed in thousands, and are means with a 95% uncertainty interval (in square brackets). ^b^ Alzheimer’s disease and other dementias. ^c^ Neurological complications due to preterm birth.

**Table 2:** Natural macamides extracted from *Lepidium meyenii* (Maca) ([16]).

Name	Formula	2D	3D (ESP)
N-benzyl-9,16-dioxo-10E,12E,14E-octadecatrienamide	C_25_H_33_NO_3_	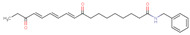	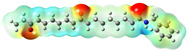
N-benzyl-16(S)-hydroxy-9-oxo-10E,12E,14E-octadecatrienamide	C_25_H_35_NO_3_	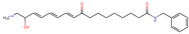	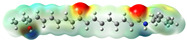
N-benzyl-5-oxo-6E,8E-octadecadienamide	C_25_H_37_NO_2_	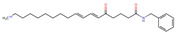	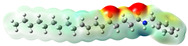
N-benzyl-hexadecanamide	C_23_H_39_NO	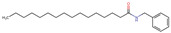	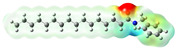
N-benzyl-9-oxo-12Z-octadecenamide	C_25_H_39_NO_2_	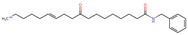	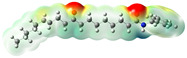
N-benzyl-9-oxo-12Z,15Z-octadecadienamide	C_25_H_37_NO_2_	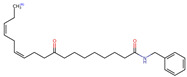	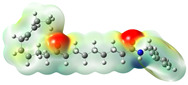
N-benzyl-13-oxo-9E,11E-octadecadienamide	C_25_H_37_NO_2_	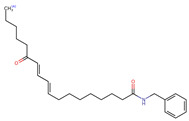	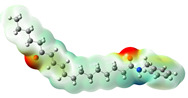
N-benzyl-15Z-tetracosenamide	C_31_H_53_NO	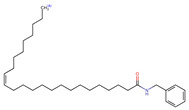	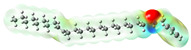
N-(m-methoxybenzyl)-hexadecanamide	C_24_H_41_NO_2_	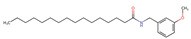	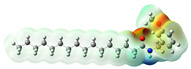
N-benzyl-9Z-octadecenamide	C_25_H_41_NO	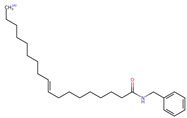	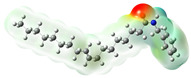
N-(m-methoxybenzyl)-9Z-octadecenamide	C_26_H_43_NO_2_	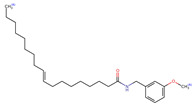	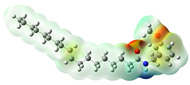
N-benzyl-9Z,12Z-octadecadienamide	C_25_H_39_NO	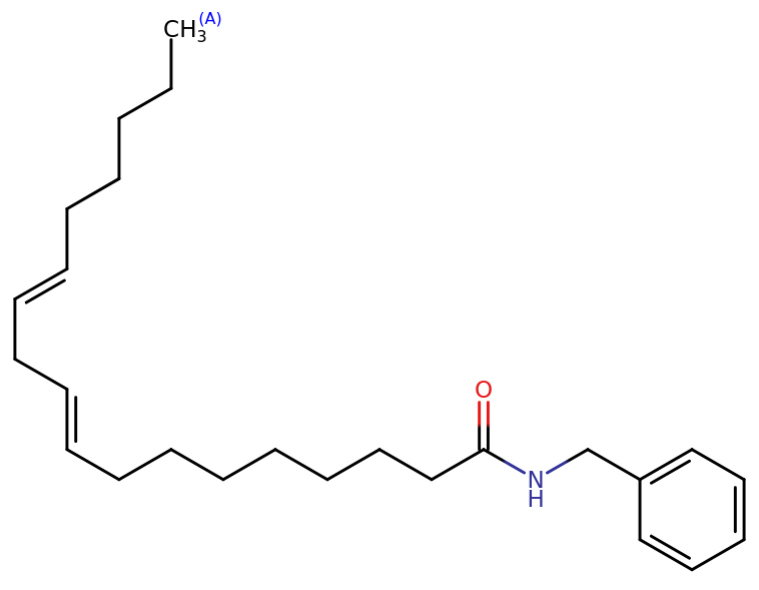	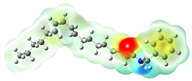
N-(m-methoxybenzyl)-9Z,12Z-octadecadienamide	C_26_H_41_NO_2_	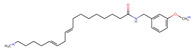	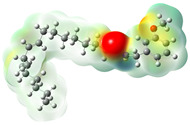
N-benzyl-9Z,12Z,15Z-octadecatrienamide	C_25_H_37_NO	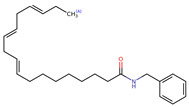	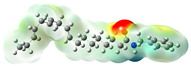
N-(m-methoxybenzyl)-9Z,12Z,15Z-octadecatrienamide	C_26_H_39_NO_2_	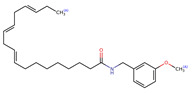	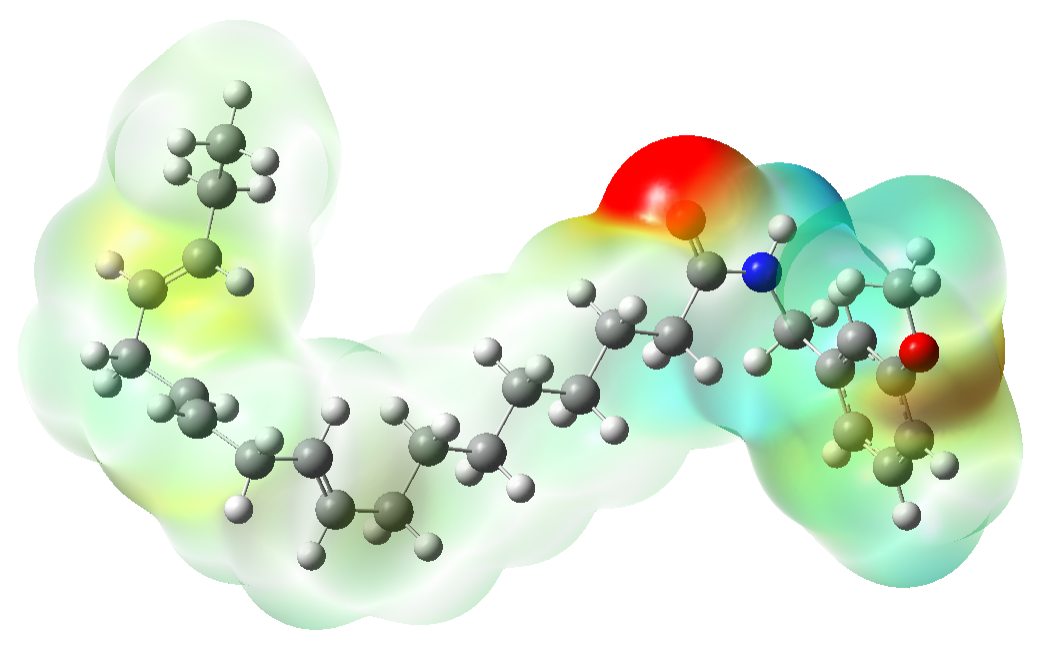
N-benzyl-octadecanamide	C_25_H_43_NO	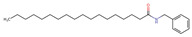	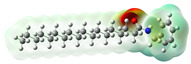
N-benzyl-pentadecanamide	C_22_H_37_NO	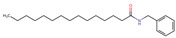	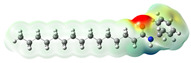
N-benzyl-heptadecanamide	C_24_H_41_NO	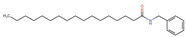	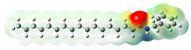
N-benzyl-octanamide	C_15_H_23_NO	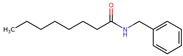	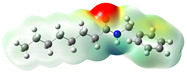
N-(m-methoxybenzyl)-octanamide	C_16_H_25_NO	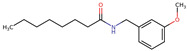	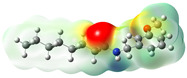
N-(3,4-dimethoxybenzyl)-hexadecanamide	C_25_H_43_NO_3_	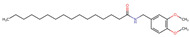	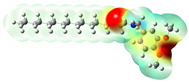
N-benzyl-tetracosanamide	C_31_H_55_NO	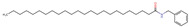	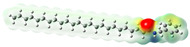
N-benzyl-2E-octadecadienamide	C_25_H_41_NO	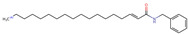	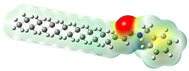
N-benzyl-9-oxo-10E,12E-octadecadienamide	C_25_H_37_NO_2_	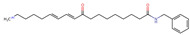	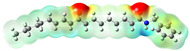
N-benzyl-9-oxo-10E,12Z-octadecadienamide	C_25_H_37_NO_2_	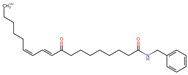	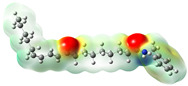
N-(3,4-dimethoxybenzyl)-9Z-oleamide	C_27_H_45_NO_3_	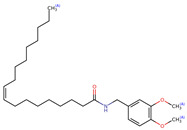	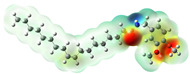

Calculated electrostatic potential surfaces on the molecular surfaces of 26 macamide compounds. The color ranges are in au, from red (−0.070) to blue (+0.070). HF/6-311G* theoretic level was used for calculations. Isodensity value = 0.004.

## Data Availability

Not applicable.
